# A device binding method based on content illumination pattern in public display environments

**DOI:** 10.1371/journal.pone.0214493

**Published:** 2019-05-10

**Authors:** Sangsik Kim, Joonyoung Park, Myungsu Chae, Sungkwan Jung, Hojong Chang

**Affiliations:** 1 Office of Academic Information Affairs, Hannam University, Daejeon, Republic of Korea; 2 KAIST Institute for IT Convergence, KAIST, Daejeon, Republic of Korea; University of New South Wales (UNSW Sydney), AUSTRALIA

## Abstract

Digital public displays installed in various locations provide valuable information for the passers-by. However, the static characteristic of the digital public display limits the consumption of the displayed content to a small area. Personal mobile devices such as smartphones are now capable of interacting with digital public displays, which enables the passers-by to “take-away” the content and consume it on-the-go. This process requires device binding, content selection, and transfer between the two devices. In this paper, we propose a device binding method which utilizes the content brightness changing pattern as a unique content ID on the public display and an illuminance sensor on the mobile to bind and transfer between two devices. We conducted performance evaluations for binding algorithm robustness in different conditions. Also comparative studies among other binding interaction methods were conducted. Our results show that our proposed method performed stably across the various conditions and overall performance in interaction completion time and error rate was similar or superior to the existing methods.

## Introduction

Digital public displays are increasingly available in the urban areas [1–5]. They are installed by service providers as large as a governmental organization to a small restaurants and retail shops. The content of the public displays span from advertisements, local events notice, to providing transportation schedules. The proliferation of such public displays along with the interactive technology including touchscreens and networking capabilities led to an environment where the content can be consumed in an interactive manner. The passersby may navigate displayed information using touch gestures and touch keyboard inputs in these interactive public displays. Recent studies attempts to bring in mobile devices to interact with the public displays, to enrich the content consumption experience [6].

We particularly consider an environment where the passersby want to “take-away” or “save” the content shown on the public display, so that he/she can consume the retrieved information in other locations. For example, the content could be an address and phone number of a local restaurant that the passerby wants to visit. It is likely that they would use the smartphone to take a photo of the displayed information. However, if the content is interactive, such as a video or a news article that they want to view on their way to another location, the content itself or the URI of the content must be transferred to their mobile device. Also as a number of researchers have pointed out that some information in the public display are prone to privacy attacks (e.g. shoulder surfing of personal email account input during a service subscription) [7, 8], mobile devices are introduced as a solution to the problem by “downloading” or “transferring” the content for personal viewing.

Despite the potential benefit of downloading the content from the public display to the smartphone, it poses several challenges. To transfer the content from one device to the other, connecting the two devices are required. It is called device association, binding, pairing or coupling [9]. There are generalized approaches such as RFID, NFC, Bluetooth or WLAN that can broadcast the public display URI to the smartphone for pairing. However, it either needs additional hardware support (e.g. RFID, NFC) or must undergo a cumbersome process of finding and selecting the target public display on the smartphone (e.g. Bluetooth, WLAN). Many researchers have tackled this problem and proposed more intuitive and efficient way of pairing using gesture, beacons and markers. However, their target device was between mobile devices. Thus it is not feasible for the static public displays.

Also downloading the content from the public display to the mobile device not only involves device binding, but also the process of selecting the content. Recent survey by Ardito *et al*. [1] shows there are substantial interaction techniques for controlling the content on the public display using personal mobile devices or gestures. Also Chong et. al [9] surveyed over a hundred device binding methods across twelve categories of binding techniques. However, most of them did not consider the pairing process independently, and assumed two interacting devices are already paired.

In this study, we developed and evaluated an illuminance sensor based device binding and interaction technique for content sharing specially designed for public display environments. We utilize the public display’s blinking signal to detect and bind the mobile device. It does not require any additional sensors other than the touchscreen on the public display side, and the illuminance sensor which is already available on any smartphones. We integrated device binding to content selection and delivering in a single interaction process in order to meet the need of the temporal interaction time that a general passerby would expect. The main research questions of this study were to 1) evaluate proposed device binding algorithm to understand the impact of various contextual conditions that may influence our proposed device binding algorithm to find appropriate variables for robustness and 2) comparatively evaluate its performance in the real-world scenarios. We conducted performance evaluations to evaluate its device binding completion time, the recognition rate under indoor/outdoor conditions as well as user’s mobile device holding postures. We also performed a comparative study among four other interaction techniques to understand the advantages and disadvantages of our proposed method in a various interaction conditions.

## Related work

Rashid *et al*. defined device binding as “a way of coupling two devices by explicitly or implicitly creating a software plus network connection between them.” [10]. Today, most common way of device binding is using wireless communication protocols such as Bluetooth, WiFi, NFC etc. Bluetooth and WiFi already provide a device discovery protocol [11, 12], enabling “scan and select” of the target device for binding. However, solely depending on these protocols require a high level of user involvement. Therefore, a large body of work has been conducted during the last decade to address this issue of simplifying user interaction process. Particularly the emergence of smartphones led to development of binding techniques based on diverse sensors such as accelerometer [13, 14], Bluetooth [15], infrared [16, 17], NFC [15, 18], RFID [19], and touchscreens [20].

### Gesture based binding

Hinckley *et al*. [13] proposed a concept of binding through synchronous gestures by bumping two tablet devices with one another to create an identical accelerometer signal on both devices. The signal data is then shared via network to be compared. If the signal matches, the server establishes the connection between them. Along with the bumping gesture as a binding method, Hinckley *et al*. also introduced display stitching technique [20] which utilizes touchscreen gesture from one device to the other. Similar to the bumping, the touch gestures on two devices are shared through the network and compared. Similarly, Nielson *et al*. [21] used pinching gestures across two display devices to establish connection. Maryhofer binded two mobile devices by holding and shaking them at the same time [22].

Bumping based binding may not be appropriate for public display—mobile interaction. Experiment results have shown that bumping mobile devices creates an inhibition due to the worry of damaging the phone [10]. Such inhibitions may be greater when interacting with public display which is a fixture that is much bigger than the personal mobile device. Also hold-and-shake type of interactions are not possible for public display interaction.

### Beacon based binding

Another type of device binding is by using beacon signals. Aumi *et al*. developed DopLink [23] which utilizes Doppler effect as an encoded sound signal. The user presses a button to initiated inaudible sound and makes a pointing gesture toward a target device. This interaction creates a change of sound frequency received on the devices within the vicinity. All devices will report their received frequency to the server. The target device will receive the maximum frequency which is then compared in the server to be recognized as the target device to be connected. Similarly, Yu and Chang used Doppler Effect to detect the target device to bind by analyzing the frequency shift among the candidate devices [24]. SurfaceLink [25] uses the natural sound created by the surface gestures to bind a set of devices placed on the same surface. Surfaces gestures such as scratching or swiping is sensed as an acoustic signal, which is then analyzed for device binding purpose.

Sound based binding has some limitations in the public settings, where the street noise could influence the robustness of the system. Also none of the work above considered multi-user interaction environments which is a critical issue for public display interactions.

Bluetooth beacon-based binding can also be used. Bluetooth Low Energy(BLE)’s initial role as a data transfer protocol has extended its role to an opportunistic device binding systems. Narzt *et al*. proposed BIBO [26], which replaces RFID tags by client(*i.e*., smartphone) BLE advertising unique ID to the vehicle information system. However, BLE based binding can be limited to the newly installed public displays, because it requires BLE receiver on the display side. It could be a burden to re-install an additional hardware for the legacy service providers.

### Camera based binding

Binding by visible codes is another way of pairing devices. One of the most well-known method is the QR-Code which is a 2D matrix code. HuddleLamp [27] uses QR-Code to bind one or more devices to their server by starting a web application. Schmitz *et al*. [28] also used a camera based binding by placing a unique marker on each device and taking a picture of the entire setup. The “phone as a pixel” system utilized phone’s color transition as a device ID. The client browses a webpage containing JavaScript application. The server then assigns a unique ID which changes the color on the mobile device display. The camera on the PC captures the device ID as well as the location coordinates of multiple devices to be a part of a larger display system.

Similarly, Parker *et al*. used mobile AR marker by embedding a unique design pattern on the background image of the public display to be recognized using the mobile camera [29].

QR-Code based binding is widely used. However, occupying a space in the public display is a problem in terms of the screen real-estate and the aesthetics. It is not different for the mobile AR maker which requires a space to encode the display’s unique ID to public display’s background design pattern. Also the QR-Code capturing gesture with the mobile device requires precise aiming of the target, depending on the size of the QR-Code. Mobile device generating a color code pattern for the camera equipped device to connect may be suitable for the public display environment. However, the color sensing camera should be on the PC (or the public display) and in the multi-user interaction environments, there is a possibility of non-relevant passers-by may obstruct the sight of the camera leading to recognition errors. Also, the user may not know the area which is capable of interaction due to the limited camera angle and code recognizable distance.

Relatively new approach of camera-based binding is by taking the photo of the display, or the appliance itself. Freitas *et al*. introduced Snap-To-It system which enables binding through image taking, and provides target appliance adaptive user interface on the mobile side [30]. Despite its convenience, it requires a massive database of the appliances that are mutually exclusive for distinction. Also the accuracy would largely depend on the captured image quality, which is not feasible in the dynamic public display environments.

## Proposed method

The key concept of the proposed method is binding two devices by matching the illumination pattern of a public display or a specific content on the display with the mobile phone’s illuminance sensor data. The user simply touches the display to bind with, or the content to be delivered to the mobile phone. Our illumination pattern based binding algorithm is employed during the simple and natural way of user interaction process. This does not require any peripheral hardware on the public display which could be applied to any general public displays in the market. The only requirement of the public display is that it is networked with a server and is touch sensitive. Since almost all smartphones are equipped with illuminance sensors, no further hardware requirement is needed.

**Fig 1:**
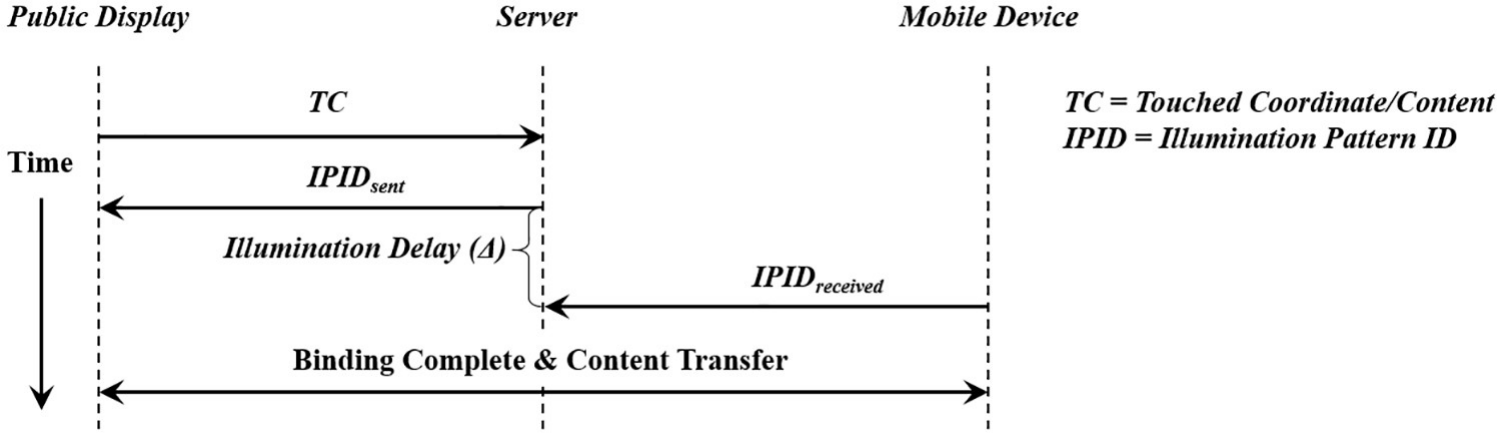
Information exchange process among public display, server, and mobile device.

Fig 1 describes the information exchange among the public display, mobile device, and the server. The user touches the display or the specific content with the mobile phone facing the public display. The touch coordinate or the touched content (TC) information is delivered to the server. This interaction is designed to facilitate the natural intention of the user to “connect my mobile device with this display” or “deliver this content to my mobile device”.

Once the server receives the TC information, it responses to the public display with an illumination pattern ID (IPID) to actuate an illumination pattern. This illumination pattern is randomly assigned from a predefined set of patterns. This pattern is a ternary number sequence consisting 0, 1 and 2 that maps with the display illumination. When the server delivers the illumination pattern to the public display, the display or the content changes its illumination by changing the display brightness or the color. For example, the displayed content may either be dimmed or brightened by changing the brightness value or simply replacing the content with the black and white colors which in turn influences the brightness value.

The illuminance sensor on the mobile phone is generally placed on the front facing side. Therefore, by touching the public display with the mobile phone facing toward the display allows the illuminance sensor to sense the brightness changing signal. This signal is processed within the mobile phone and decoded into the original IPID. The mobile phone sends this IPID to the server, and the server maps this ID with the display or the content URI within the mapping table. Once the server finds the match, server either binds them by establishing a connection between two devices or delivers the content URI to the mobile device. The illumination pattern generation and recognition algorithm is further described in detail in the next section.

### Illumination pattern ID (IPID) assignment

When the mobile device touches the display or a specific content, the public display delivers the URI of the touched content (TC) to the server. At the same time, the public display receives the content illumination pattern ID (IPID) from the server and change the brightness of the touched content area. The IPID is an n-bit ternary number sequence which consists of 0, 1 and 2. This IPID is defined as follows.

$$S=\{\sum_{0}^{n-1}\alpha_{i}\times 10^{i}\;|\;\alpha_{i}\in \{0,1,2\},\alpha_{n-1}=0,\alpha_{n-2}=2\}$$ (1)

The server assigns a random IPID for the corresponding content (TC) by mapping the IPID to the TC. In order to allocate more than one IPID due to a simultaneous multi-requests, the server scans the mapping table to assign an available IPID for the requested TC.

### Encoding of the IPID

**Fig 2:**
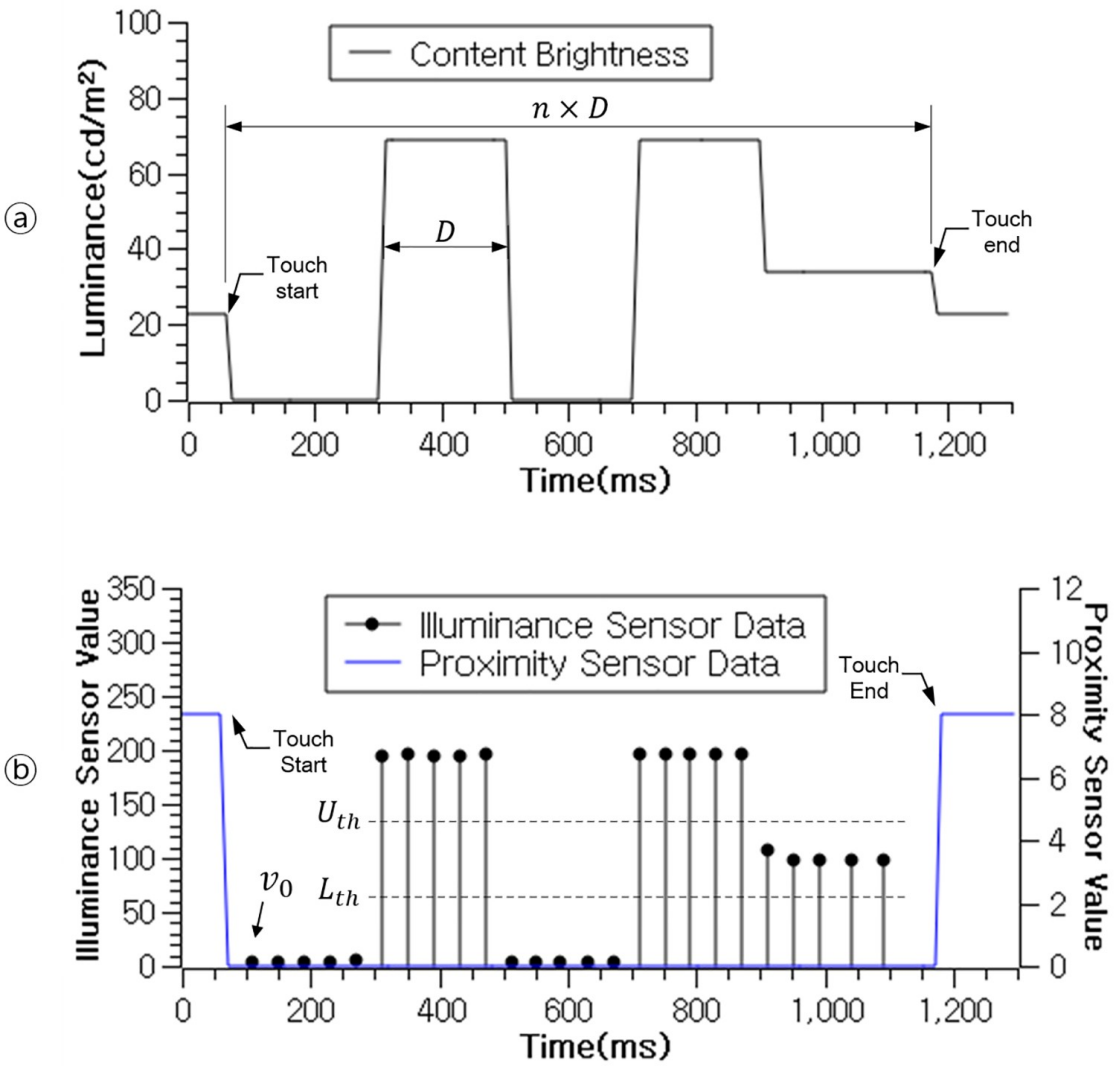
Encoding of illumination pattern ID. ⓐ: content brightness change interval and its value; ⓑ: illuminance sensor value according to the content brightness change.

The public display encodes the received IPID from the server by changing the brightness of the TC from the most significant digit (*α*_*n*−1_) to the least significant digit (*α*_0_). This illumination pattern has a “blinking” interval D, which changes the brightness of the TC. The brightness is assigned by the IPID value from the brightest if *α*_*i*_ = 2, 50% brightness if *α*_*i*_ = 1, and 0% brightness if *α*_*i*_ = 0. Fig 2 shows an example of the brightness change value of a TC when the IPID is “02021”.

### Mobile side decoding of the illumination pattern

The mobile device uses the illuminance sensor to decode the illumination pattern. We add one more step prior to the decoding of the illumination pattern as a determinant for initiation. Therefore, we describe a two phase decoding process in the following section.

#### Interaction intention recognition

Our core approach is detecting the illumination pattern of the public display using the mobile phone. However, if there are multiple mobile phones within the proximity, all mobile phones must consistently detect and transfer the illuminance sensor data to the server. Since the illuminance sensor can be easily influenced by user’s physical activities or the environment, such as the turning the room light on and off, a systematic determinant for declaring the starting point of the sensor data decoding is required. Thus we employed proximity sensing in combination with the illuminance sensing method for recognizing the intended binding interaction.

Proximity sensor is able to detect approximate distance of a nearby object. When the proximity sensor detects any object is within the predefined range (in our case less than 3cm), and the illuminance sensor detects a brightness above a certain threshold, we can infer that the mobile phone is nearly touching a light source such as the display. Therefore, when the user touches the public display with the mobile phone for binding, the proximity sensor value would return “true”. At the same time, the touched content would signal the illuminance sensor by changing its brightness value from dark to bright, which the two most significant digits would be “02”. Elsewise, the mobile device determines the binding gesture is not executed.

#### Decoding the illumination pattern

In order to decode the illumination pattern, we assume the mobile phone is given the length of the IPID *n*, illumination interval *D*. When the proximity sensor data value changes to 0, the mobile phone monitors the illuminance sensor data for a time period *n* × *D*. When the illuminance sensor data is collected m times, we can represent the retrieved illuminance sensor data as $V={\left(v_{i}\right)}_{i=0}^{m-1}$. Also the timestamp of retrieved sensor data is represented as $T={\left(t_{i}\right)}_{i=0}^{m-1}$. Therefore, we can decode the illumination pattern with the value V and T by applying the following algorithm.

- *n* indicates the length of IPID
- *D* indicates interval of changing content brightness
- *m* indicates total number of samples of illuminance sensor for *n* × *D* duration
- *v*_*i*_ indicates a value of *i*′*th* sample of illuminance sensor
- *t*_*i*_ indicates a timestamp of *i*′*th* sample of illuminance sensor
- *V*_*max*_ indicates *MAX*{*v*_*i*_ | *i* = 0‥*m* − 1}
- *V*_*min*_ indicates *MIN*{*v*_*i*_ | *i* = 0‥*m* − 1}
- *U*_*th*_ indicates two-thirds of (*V*_*max*_ − *V*_*min*_) plus *V*_*min*_
- *L*_*th*_ indicates one-third of (*V*_*max*_ − *V*_*min*_) plus *V*_*min*_
- $f(v_{i})=\{\begin{array}{ll} 2, & if\;v_{i}>U_{th}\\ 1, & if\;L_{th}<v_{i}<U_{th}\\ 0, & if\;v_{i}<L_{th} \end{array}$

**Algorithm**

**Input**: *n*, *D*, *V*[*v*_0_, …, *v*_*m*−1_], *T*[*t*_0_, …, *t*_*m*−1_]

**Output**: decoded IPID

1: getDecodedIPID(*n*, *D*, *V*, *T*);

2: **Begin**

3:  string decodedIPID← “”;

4:  long prevEdgeTime←*t*_0_

5:  double *V*_*max*_ ← *MAX*{*v*_*i*_|*i* = 0‥*m* − 1};

6:  double *V*_*min*_ ← *MIN*{*v*_*i*_|*i* = 0‥*m* − 1};

7:  double $U_{th}\leftarrow \frac{2}{3}(V_{max}-V_{min})+V_{min}$;

8:  double $L_{th}\leftarrow \frac{1}{3}(V_{max}-V_{min})+V_{min}$;

9:  **for** each *v*_*i*_ = *v*_1_ to *v*_*m*−1_ **do**

10:   **if** *f*(*v*_*i*_) ≠ *f*(*v*_*i*−1_) **then**

11:    **while** *t*_*i*_ − prevEdgeTime ≥ $\frac{1}{2}D$

12:     append *f*(*v*_*i*−1_) to the decodedIPID;

13:     prevEdgeTime + = *D*;

14:    **end while**

15:    prevEdgeTime←*t*_*i*_

16:   **end if**

17:  **end do**

18:  **while** the length of decodedIPID < *n*

19:   append *f*(*v*_*m*−1_) to the decodedIPID;

20:  **end while**

21:  return decodedIPID;

22: **end**

The proposed algorithm calculates *U*_*th*_ and *L*_*th*_. And then it computes duration(*t*_*i*_ − *prevEdgeTime*) when *f*(*v*_*i*_) ≠ *f*(*v*_*i*−1_). Then this duration value is compared with *D* for inferring the IPID received from the server.

### Server side IPID matching and binding

The server receives the decoded value of the IPID from the mobile. First, the server scans the IPID mapping table if the received IPID exists. If the server is able to match the IPID within the table, and the time interval between the IPID_*sent*_ and IPID_*received*_(Fig 1) is within a predefined threshold, it binds two devices together. This time interval is named illumination delay, which is mainly caused by the illumination pattern. If this illumination delay is within a predefined threshold, the server infers the mobile phone interacted with the public display, thus binds and transfers the content URI.

If the illumination delay exceeds the predefined threshold, the server dislocates the reserved IPID, making the IPID available for other devices.

### Support for simultaneous multi-user interaction

Proposed method discriminates each individual user interactions by allocating different IPID on each TC even if they are within the same display. This is critical for a large public display interaction system where multiple passers-by want to bind and retrieve content from the display. We have considered this simultaneous multi-user interaction into our system to be evaluated in the later section.

The number of simultaneous user is dependent on the number of IPID set. And if the length of the IPID is *n*, the maximum capability of simultaneous interactions would be 3^n^. However, since we reserved the two most significant digits as “02” for the interaction intention recognition, the actual capability of simultaneous interactions would be 3^*n*−2^. If the simultaneous interactions exceed this capability, the server waits until the IPID to be released from previous interactions. Also the length of the IPID n could be increased for a higher number of simultaneous interaction support, however it could increase the illumination delay. This issue is also further investigated in the later sections.

## Performance evaluation

Our system involves not only the software algorithm but also the hardware system, user interactions, and the environmental factors in which the interactions are performed. They all affect the performance of the system. Therefore, we conducted a multi-perspective evaluation of the proposed system.

First, the most critical evaluation point is the illumination interval and recognition rate. Evaluation for minimizing interaction delay without compromising recognition rate was conducted. Second, mobile device touch gesture angle and recognition success rate was evaluated. This is to understand how much performance is guaranteed with the different touch gestures performed with the mobile device.The illumination pattern would be more clearly sensed by the mobile device if the mobile device is facing down toward the public display. However, in the actual interaction, the angle would be greater than 0. Third, the light intensity of the environment where the interaction takes place would also influence the recognition rate. Thereby we evaluated the recognition rate by varying the ambient light intensity in the interaction environment. Lastly, we performed a comparative evaluation with other binding methods, both with single user and multiple user environments.

### Effect of illumination interval on the recognition rate

Minimizing interaction time is crucial in the general user interaction perspective. Moreover, since our system targets the “on-the-go” use case, the importance of the interaction time is emphasized. The illumination interval of the target content consumes most of the interaction time. If the illumination interval is too fast, the illuminance sensor would not be able to correctly receive the brightness change. Therefore, we varied the illumination interval to evaluate the recognition rate.

**Fig 3:**
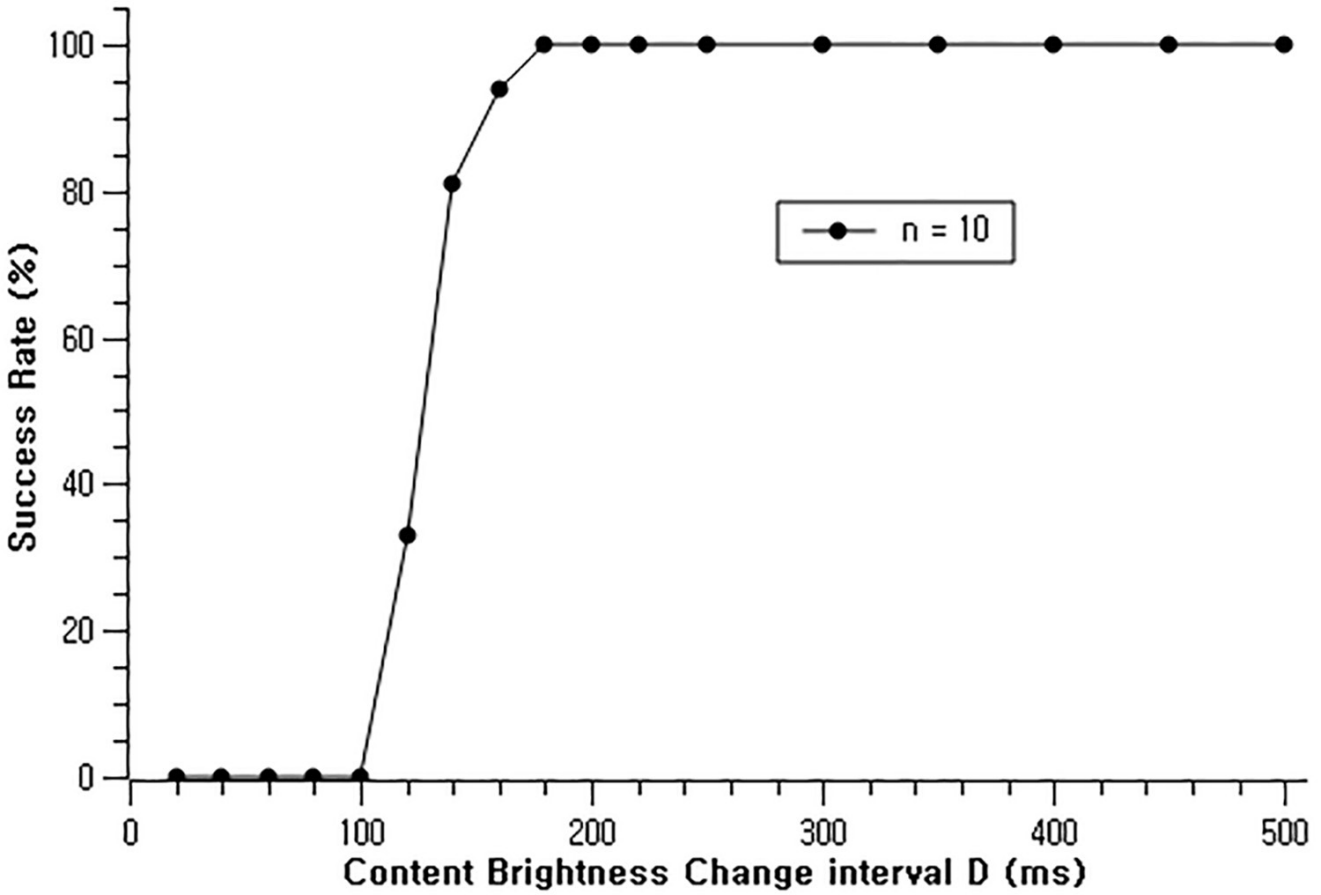
Illumination interval and the recognition rate.

We performed evaluation in the corridor of the office where the ambient light was measured 84 lux. The digital signage display was a 46” TV (Samsung SM3452) and the mobile device was Samsung Galaxy S7. The digital signage display brightness was 63*cd*/*m*^2^. We performed 100 repeated measures by varying the content brightness changing interval from 20*ms* to 500*ms*. The IPID length *n* = 10 was used for the evaluation. When interval *D* = 160*ms*, we achieved 94% recognition rate, and reached 100% when *D* was greater than 180*ms* (Fig 3).

The shorter the length of the IPID *n* is, the shorter total interaction time would take. However, the number of distinguishable IPID would also be less. The IPID length *n* can accommodate 3^*n*−2^ number of simultaneous users for device binding and content distribution. Therefore, choosing an appropriate IPID length *n* is totally dependent on the service environment and the service provider. Foundational researches of Shneiderman [31] and Miller [32] shows that users perceives the system as responsive only if the feedback is observed within 2 seconds of user input. Therefore, in order to accomplish binding within 2 seconds, the maximum length of IPID would be 11. This denotes our system is capable of 3^9^ = 19, 683 simultaneous users without compromising binding accuracy.

### Effect of device angle on recognition rate

Proposed method requires user to perform a touch gesture on the public display with their mobile device. Most of the users have no problem with this gesture, however there are individual differences in the touch angle when the touch is performed. Touch angle between the public display and the mobile device affects the binding success rate because the illuminance sensor embedded on the mobile device may or may not be able to receive enough illumination changing signal from the display. If the angle between the public display and the mobile device is greater than 90 degrees, the sensor would not be able to detect the illumination changing signal, which is the IPID. Therefore, in this evaluation, we varied different angles to measure the recognition rate.

**Fig 4:**
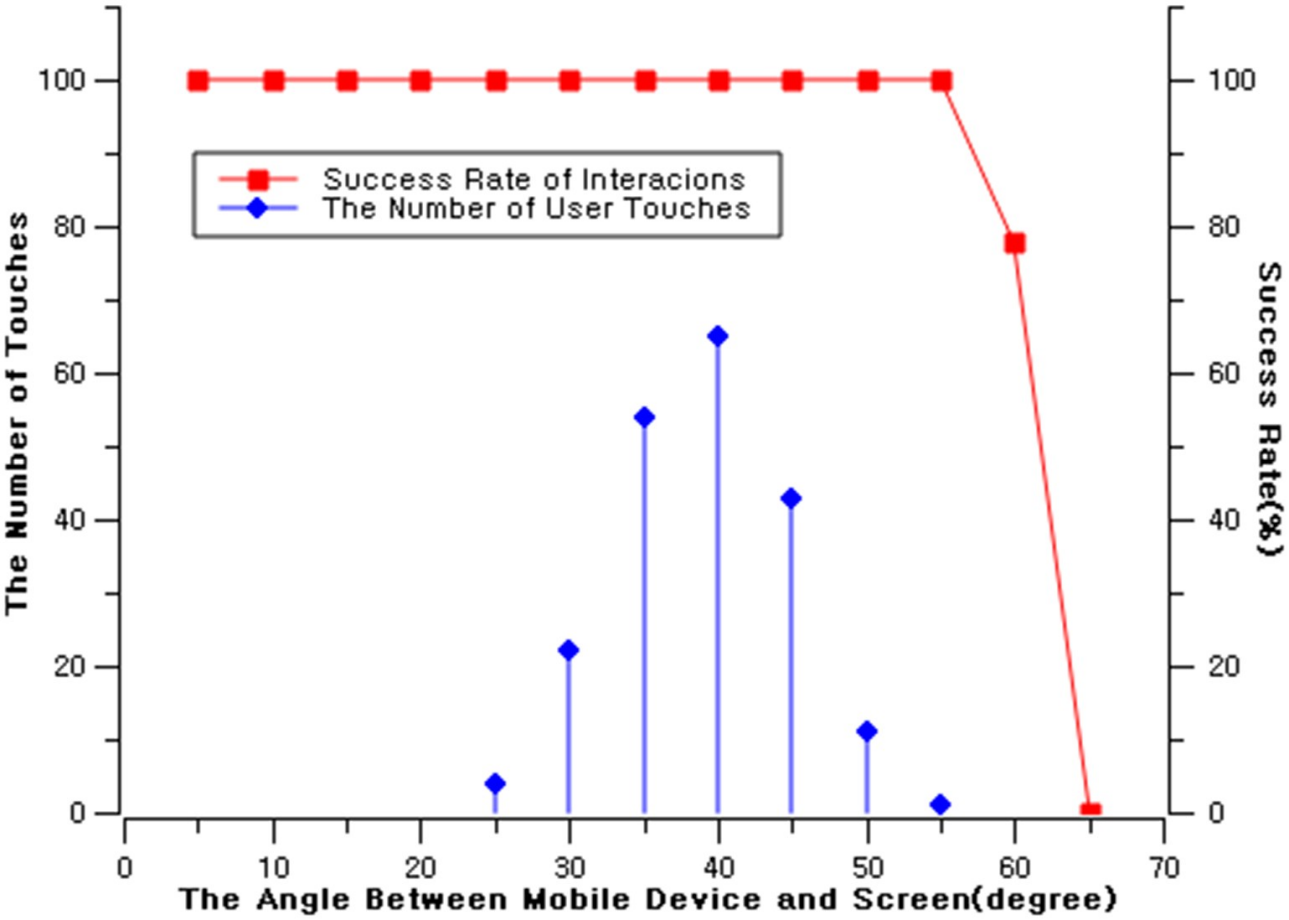
The angle measurement of mobile device and digital signage, and their success rate.

We recruited 20 participants (13 males, 7 females; mean age = 24.4, sd = 4.1) by online recruitment posting from a large university. The participants were researchers and students from various backgrounds (e.g. engineering/non-engineering), and had no particular experience interacting with large screen displays. They were well informed of the task procedures in both verbal and written descriptions. We conducted 10 repeated measures with 20 people, resulting in a total of 200 trials. The participants were asked to perform the interaction and the angle between the mobile device and the public display was measured manually using protractor. We transformed individual angles into a categorical value of every 5 degrees (Fig 4). The results show that the angles varied from 25 degrees to 55 degrees (mean = 38.9, sd = 5.8). The data was anonymized for analysis, because no personal information was needed. The task lasted 5 minutes per participant, and they were compensated with approximately 5 US dollars each. All evaluations involving human subjects in this study are all approved by KAIST with an IRB grant number KH2017-27.

Based on our initial measurements, we conducted a lab measurement for angle-recognition rate analysis. We conducted 100 repeated measures from 5 degrees with an incremental of 5 degrees until the recognition rate was 0%. The recognition rate was 100% until 55 degrees, 78% at 60 degrees.

Above two studies reveal that users’ touch generally does not go beyond 50 degrees, which is under the safe zone of less than 55 degrees. Therefore, we conclude our binding method would not suffer from sensor data retrieval errors from the physical user interactions.

### Effect of ambient light intensity on recognition rate

Our proposed method uses illuminance sensor to acquire the brightness change of the target content on the public display. Thus, the ambient light intensity in the interaction environment affects the recognition accuracy of the illuminance sensor. This includes the reflection from the display screen. In this section, we describe an ambient light intensity-recognition accuracy evaluation.

We controlled two display brightness (63*cd*/*m*^2^ and 143*cd*/*m*^2^) and varied eight types of ambient light intensities [Indoor(Dim Light) = 45 lux; Indoor(Hallway) = 111 lux; Indoor(Fluorescent Light) = 206 lux; Indoor(Office) = 415 lux; Outdoor(Rainy) = 693 lux; Outdoor(Overcast) = 1,374 lux; Outdoor(Sunny) = 12,402 lux; Outdoor(Direct Sunlight) = 33,709 lux] with a total of 16 conditions. We also varied 3 types of touch angles (10, 30, 50 degrees). The two displays used were Samsung TV SM3452 (63*cd*/*m*^2^) and Samsung TV DM48E (143*cd*/*m*^2^).

**Fig 5:**
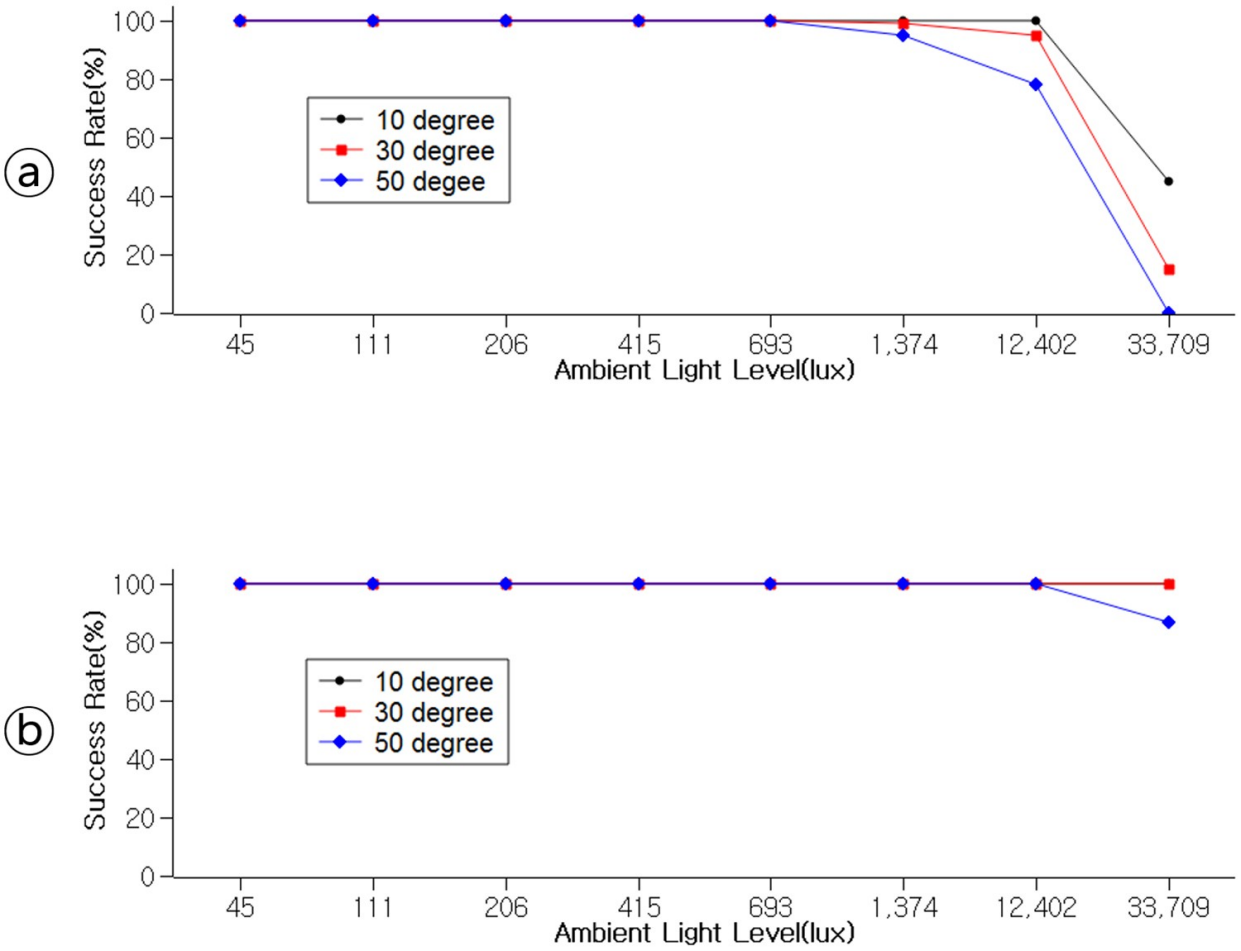
Device binding success rate under varied ambient light intensity with ⓐ: 63*cd*/*m*^2^ display; ⓑ: 143*cd*/*m*^2^ display.

As shown in Fig 5, the proposed method guarantees accurate performance in all indoors conditions. However, in outdoor condition when sunny or even under direct sunlight, the accuracy falls steeply in the darker display of 63*cd*/*m*^2^. However, the accuracy is maintained at over 87% in the brighter display of 143*cd*/*m*^2^. The displays used were not specialized for the outdoor use and were relatively dark compared to the outdoor digital signage displays with high brightness diodes (>450*cd*/*m*^2^). Therefore, we expect the recognition rate would not be compromised even under direct sunlight if applied to the outdoor digital signage displays.

## User evaluation

**Fig 6:**
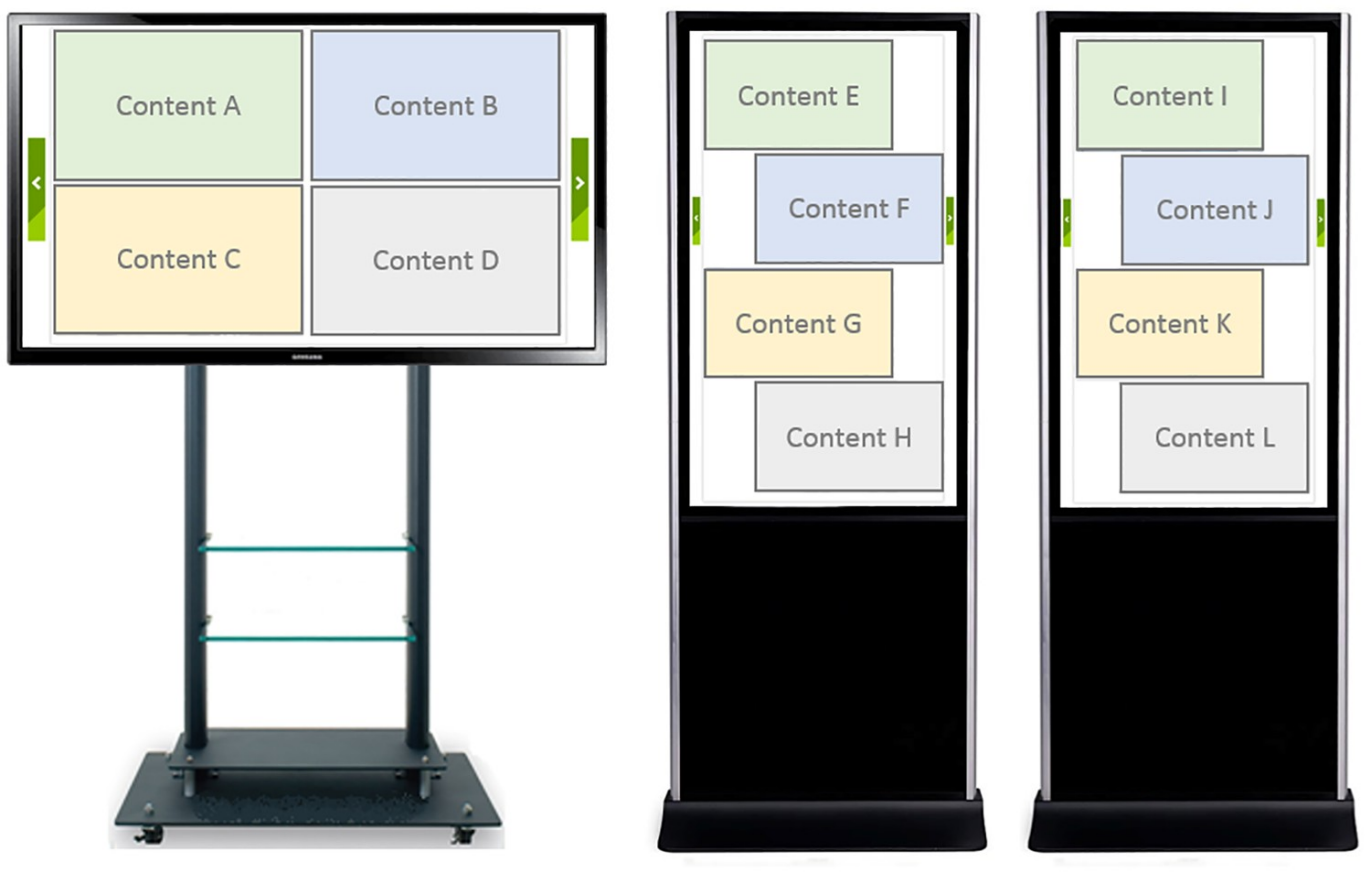
The arrangement of digital signages and the contents for evaluation.

We conducted a comparative study with existing interaction methods of acquiring content from the public display to the mobile phone including the binding process. We compared our proposed method with four other methods including 1) C-type (Camera based), 2) N-type (NFC based), 3) A-type (Accelerometer based), 4) B-type (Beacon based). We measured the interaction completion time and error rate in each interaction. Three public displays were placed in the lab which rendered four types of content as shown in Fig 6. For all interaction methods, we measured the completion time from the point when the user presses “start” button on our experiment application until the target URI is delivered to the mobile device. The participants were exactly 1.5 meter away from the public display, where the public display’s content is visible.

First, camera based interaction is a way of acquiring content from another device. The most representative example is by using QR-codes. This requires the user to target the camera’s view angle using the mobile device toward the QR-code embedded on or beside a content.

Second, NFC based interaction is a way of binding the mobile device with a public display. The user first touches and selects the content to transfer on the public display. Once the content is selected, the participant touches the NFC tag placed at the bottom corner of the public display. The selected content URI is then delivered to the mobile device.

Third is the accelerometer based interaction. Similar to Bump [13], the participant touches the public display content with the mobile device. The touch creates an accelerometer signal on the mobile device and the touch coordinates on the public display. They are sent to the server at the same time, binding the two device and delivering the content URI to the mobile device. However, unlike Bump [13], we did not embed accelerometer in the public display side. Because we wanted to maintain the fact that the public display does not need to install additional sensors. Therefore, the recognition rate of our setup is lower than the original Bump approach.

Fourth is the beacon based interaction. The display is equipped with a WiFi beacon, broadcasting all the content to the mobile devices available within the vicinity. When the mobile device detects a certain WiFi AP, it renders the content list as a thumbnail similar to the work by Kim *et al*. [33]. Therefore, the mobile device rendered 12 types of content at the same time. The user had to find the target content and touch-select for the evaluation.

In order to evaluate the performance of each interaction types in different conditions, we setup an experimental environment in 3 scenarios: 1) single user interaction, 2) multiple user simultaneous interaction, 3) video content interaction. 46” display (SM3452) was used as the digital signage, and Samsung Galaxy S7 was used for the mobile device. The experiment was conducted in an office environment under the ambient light brightness was approximately 415 lux. The hardware and environmental settings were the same across three scenarios.

### Scenario 1: Single user interaction

**Fig 7:**
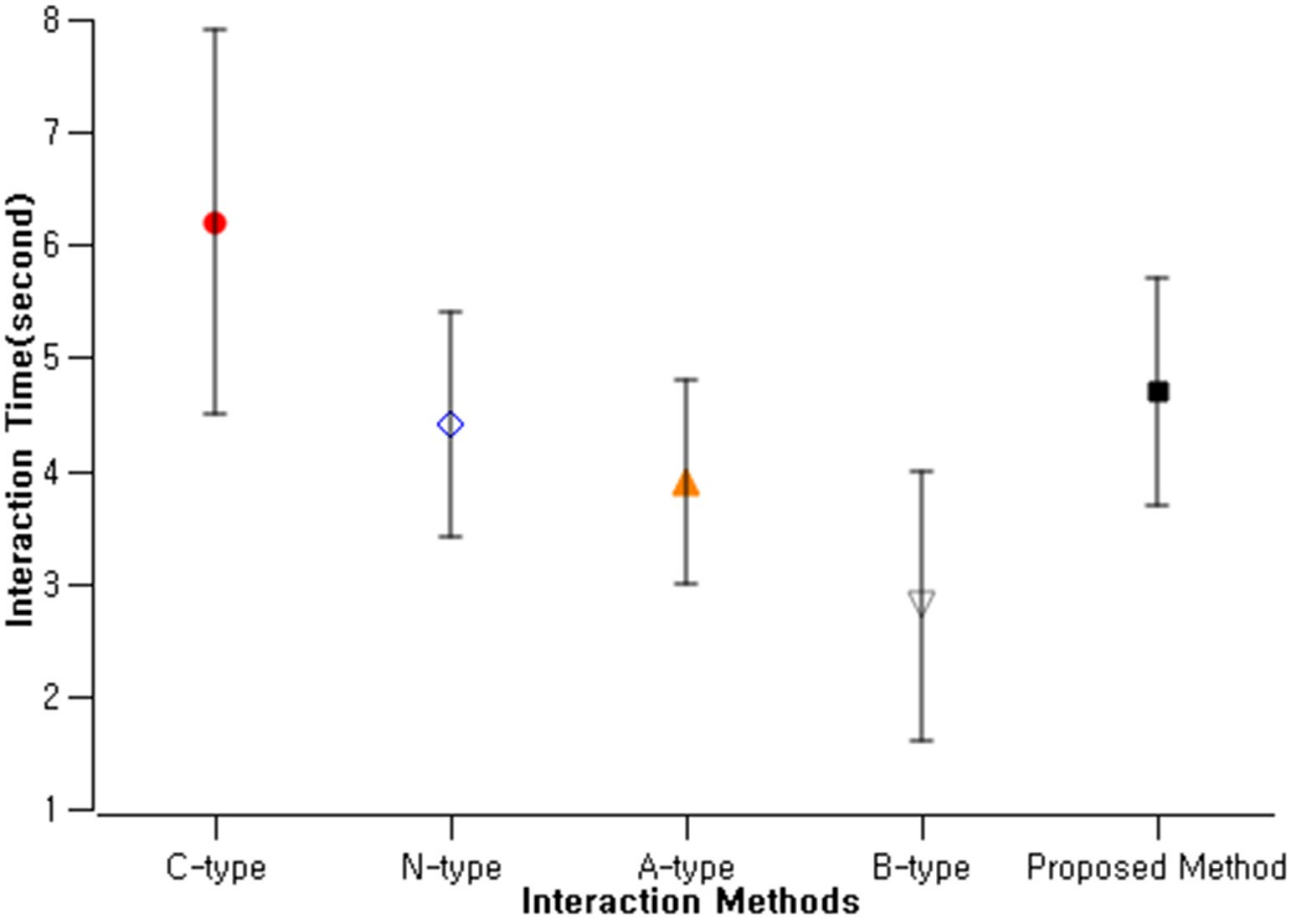
Interaction methods and their interaction completion time.

This scenario is the simplified interaction scenario where only a single user is present in front of the public display and interact with a content to be delivered to his mobile device. The content was composed of static image and text. Each participant was asked to stand on the starting line which is 1.5m away from the public displays. A total of 24 participants (mean age = 25.3, sd = 5.0) were recruited. They were asked to collect the target content in the digital signage display using 5 different types of interaction methods, and their interaction time was measured (Fig 7). In order to minimize learning effect and ordering effect, we counter-balanced the order of the interaction methods using Latin Square, yielding 120 combinations. Each participant were asked to complete a total of 10 interaction combinations. This allowed us to collect a total of 1,200 interaction trials.

Across all interaction types, the users were able to finish the interaction between the average of 2.8 to 6.2 seconds (Table 1). B-type was the fastest and C-type was the slowest. This was mainly because B-type did not require the user to approach the public display and acquire the content at the standing position. On the other hand, we could observe some difficulties from the participants in the QR-code capturing process such as the holding and aiming the camera to focus on the QR-code. We initially predicted that the QR-code would be overall the most well accepted interaction type, but unevenly distributed completion time in our results shows several users had difficulty in doing so.

**Table 1:** Interaction methods and their interaction completion time (in seconds).

	C-type	N-type	A-type	B-type	Proposed Method
**Mean**	6.2	4.4	3.9	2.8	4.7
**SD**	1.7	1.0	0.9	1.2	1.0

### Scenario 2: Multiple user simultaneous interaction

**Fig 8:**
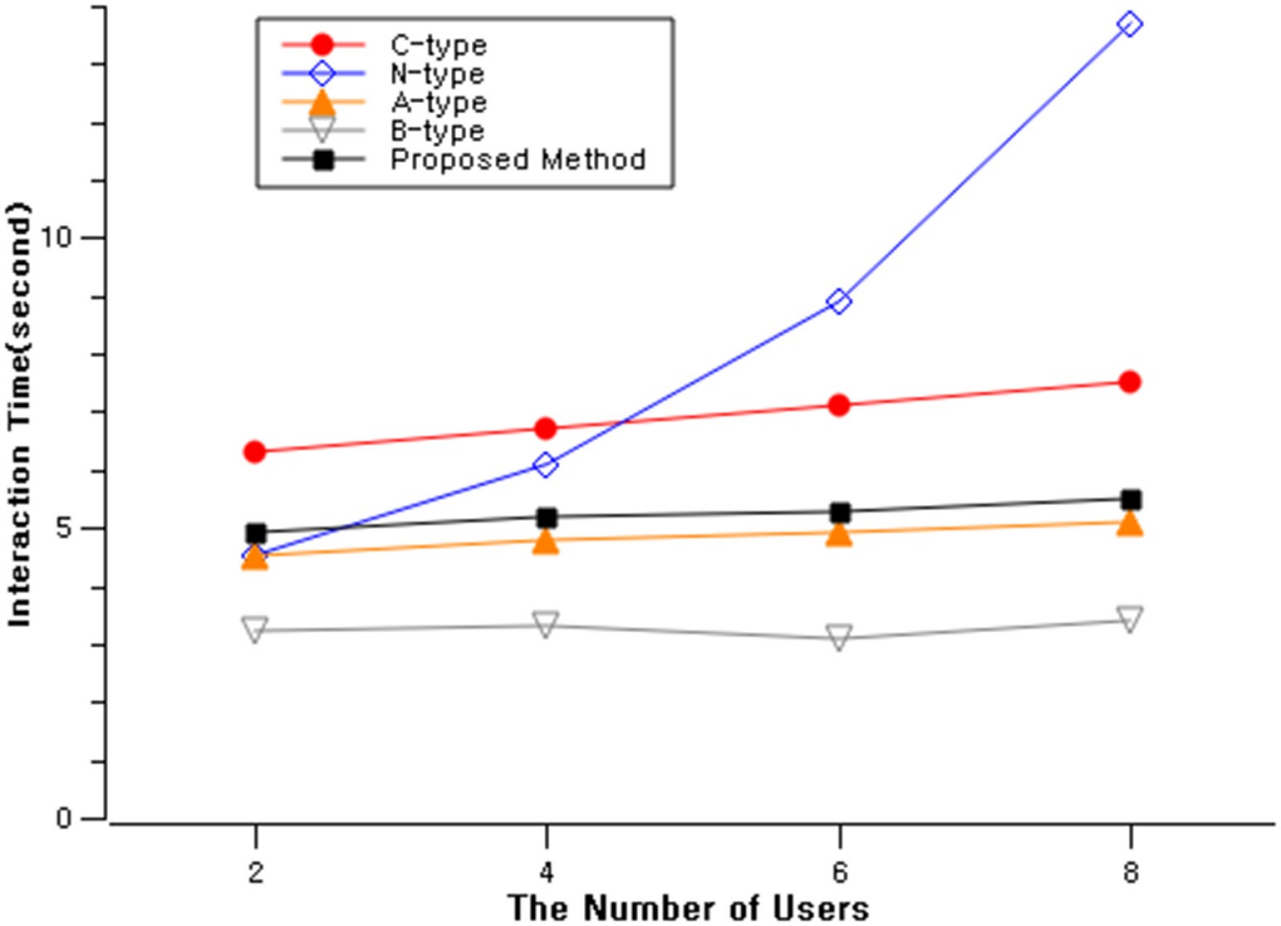
Comparison of interaction types in completion time and simultaneous users.

In this scenario, multiple users exist within the vicinity and interacts with the public displays. They were given the same 5 types of interaction methods, same as scenario 1. We recruited 20 participants(mean age = 24.2, sd = 3.5), different from the participants from scenario 1. 20 participants were randomly assigned to one of the four groups (2/4/6/8 people in a group) and each group was assigned with one of the 5 types of interaction methods. Their interaction time was measured for each interaction methods with four different groups (Fig 8).

Interaction completion time across all types did not change much except for N-type, which showed a dramatic increase from average of 4.5 seconds to 13.7 seconds (Table 2). This was due to the fact that the number of NFC tags were less than the number of simultaneous users. Therefore, the participants had to line up and wait for the previous user’s interaction to be finished. Therefore, N-type was not appropriate for the simultaneous user interacting environments.

**Table 2:** Comparison of interaction types in completion time and simultaneous users (in seconds).

Mean (SD)				
	2-users	4-users	6-users	8-users
**C-type**	6.3(1.6)	6.7(1.7)	7.1(1.7)	7.5(1.9)
**N-type**	4.5(0.8)	6.1(2.6)	8.9(3.4)	13.7(7.2)
**A-type**	4.5(0.9)	4.8(0.9)	4.9(0.9)	5.1(1.0)
**B-type**	3.2(1.4)	3.3(1.1)	3.1(1.5)	3.4(1.3)
**Proposed Method**	4.9(0.9)	5.2(1.1)	5.3(0.9)	5.5(1.0)

**Fig 9:**
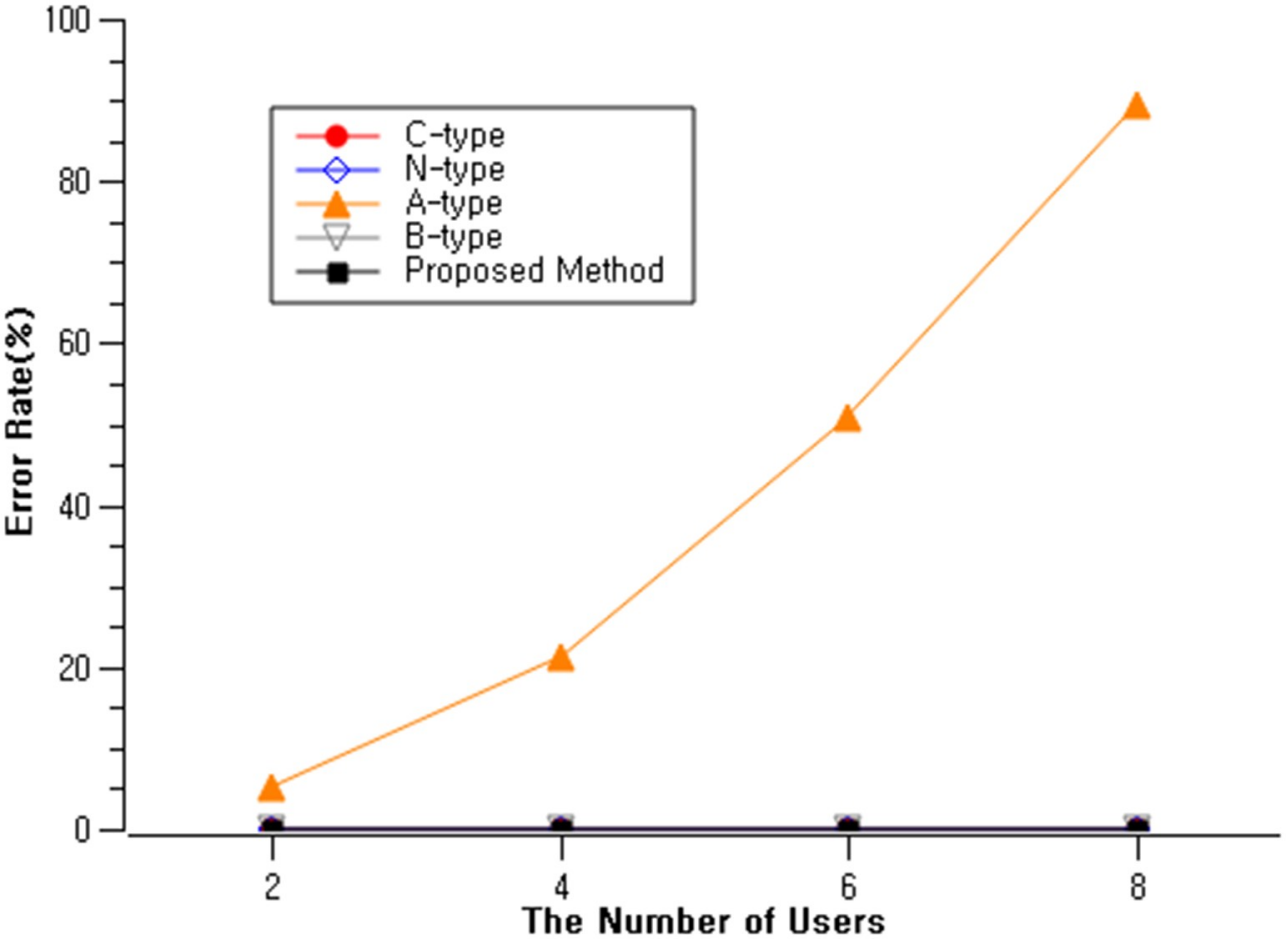
Comparison of interaction types in error rate and simultaneous users.

The error rate from the above trials were measured (Fig 9). All interaction types showed 0% errors except for A-type. The accelerometer based interaction showed an error rate of 5.0% when two simultaneous users were present, and up to 89.4% when eight users interacted. This was due to the following reasons. First, lack of distinguishable amplitude of the accelerometer data. Some users bump the display too lightly, but in order to make sense of the bumping gesture as the public display touch intention, we need a greater accelerometer value. Second is the inability to distinguish between the users. There is no way of distinguishing those two interactions if two touches were simultaneously performed on the same public display. The probability of errors increase as the number of interacting users increase. Third, meaningless movements of the mobile devices are mixed up. There could be some users holding the mobile device ready, but not yet touched the public display. Their mobile device movement data are transferred to the server real-time, which then could mislead to be included in the target device candidate. Therefore, A-type is not appropriate for multiple user interacting environments.

### Scenario 3: Video content interaction

**Fig 10:**
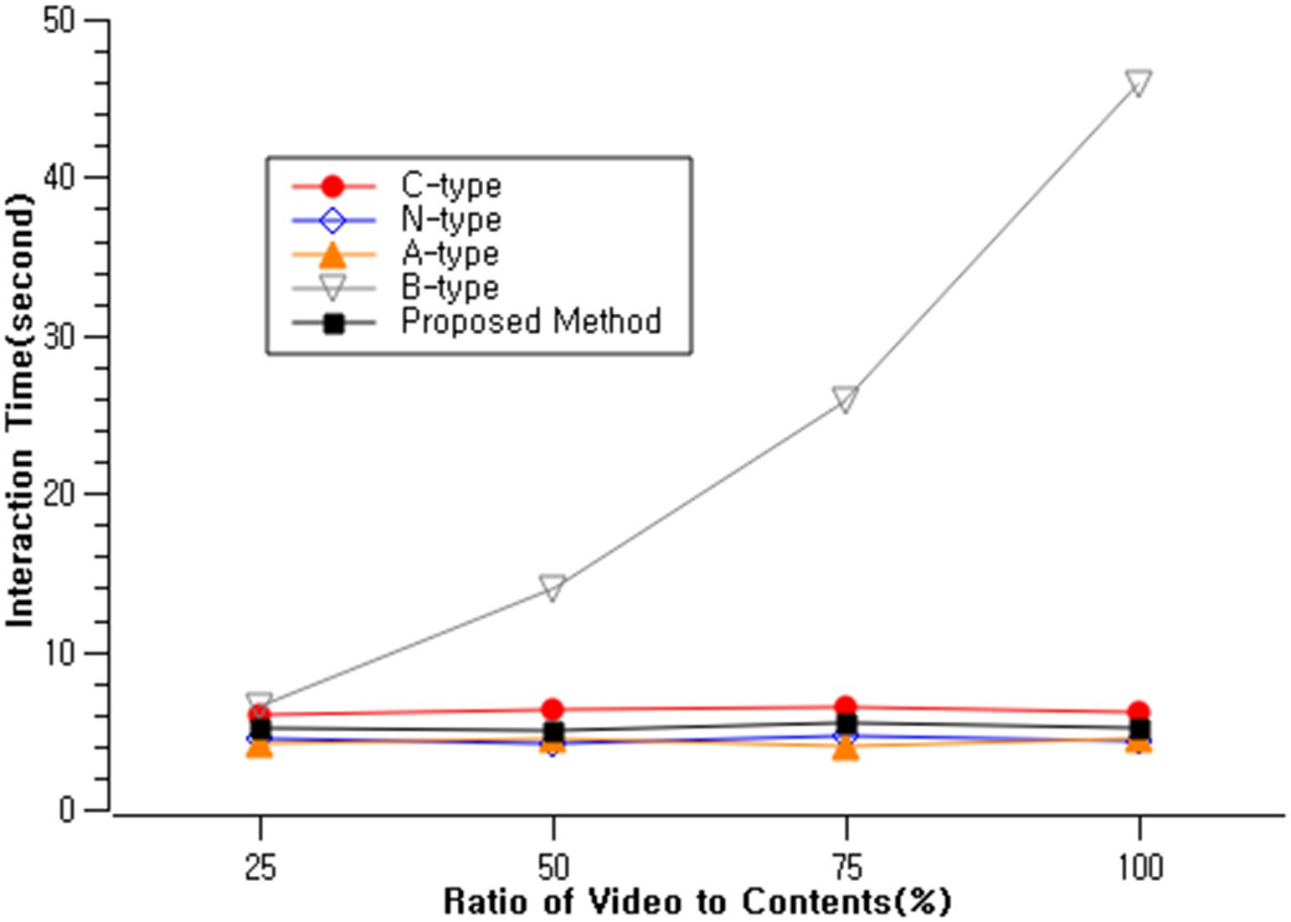
Comparison of interaction types in completion time with video contents.

Digital public displays are capable of rendering text, image as well video. In this scenario, we further explore how the different types of interactions are performed with video contents. We varied the number of video content rendered on the public displays. Among the 12 content slots, we located 3, 6, 9, and 12 video contents across the public displays. This was similar to the single user interaction scenario, with four additional displayed content types. We recruited 20 participants(mean age = 25.8, sd = 3.4), different from the participants from scenario 1 and 2. This yielded a total trial of 4,800 (20 participants × 5 interaction methods × 12 counter-balanced trials × 4 content types). Their interaction time was measured for each interaction methods with four different video content ratios (Fig 10).

Our results show that all interaction types’ completion time were consistently between 3.9 and 6.5 seconds (Table 3). However, beacon based interaction showed a dramatic increase in completion time as the ratio of the video increased. This was due to the fact beacon based interaction requires the user to go through “search and select” process with the mobile device. It is notable that the beacon based interaction showed similar interaction completion time with other interaction types in scenario 1 and 2, where we used still image contents. However with videos, the content shown on the public display is dynamic, changing its image frame during the interaction process. When the 12 contents on the public display is displayed on the mobile side, the participants need to go execute a mental search process to match the video between the public and mobile displays. The results show that this is not an easy process, particularly when all the contents are videos, it took approximately 10 times the completion time of other interaction types.

**Table 3:** Comparison of interaction types in completion time with video contents (in seconds).

Mean (SD)				
	25%	50%	75%	100%
**C-type**	6.0(2.0)	6.3(2.0)	6.5(2.3)	6.2(1.8)
**N-type**	4.4(1.1)	4.1(1.1)	4.6(1.1)	4.3(1.1)
**A-type**	4.2(1.0)	4.4(1.1)	3.9(1.1)	4.4(1.1)
**B-type**	6.5(5.0)	13.9(11.4)	25.9(16.1)	45.8(18.3)
**Proposed Method**	5.1(1.1)	4.9(1.2)	5.4(1.2)	5.2(1.1)

## Discussion

The performance of the proposed algorithm is heavily dependent on the systematical parameters of illumination interval, environmental parameters of ambient light intensity, and user interaction parameters of device angle between the public display and the mobile device. We revisit each performance evaluation results in accordance with system and user interaction design implications.

### Implications for system design

We focused on the detailed performance evaluations under varying system/environmental conditions for the practitioners to replicate our work and given customize the parameters to their needs. Through our analysis, we emphasize the feasibility of the algorithm in the real-world context. The varying illumination interval recognition results show that the illumination interval greater than 180ms reached 100% recognition rate. This may serve as a pivot point for the trade-off between recognition rate and binding delay. Considering there needs to be multiple illuminations to encode a content ID, an appropriate delay time can be implemented to suit the service context. For example, during a crowded busy morning at a point of transit (POT), the service provider may want to give up some recognition rate for a shorter delay. In the context of point of wait (POW) in the restaurant, the delay may not seem to critical, thus keep the illumination interval above 180ms. Our analysis with ambient light provides insights in the display brightness requirements and how the display should be placed in the outdoors. Display brightness capability is increasing every year, and the recent outdoor digital signage is capable of 5,000*cd*/*m*^2^, which is way above our experimental condition. This implies our algorithm would be much more robust as the display technology grows.

### Implications for interaction design

Our integrative approach of “inter-device binding” and “content selection and transfer” differs from prior works that separately manages these two processes. For example, our method uses direct contact with the content using the device reduces the two-step process into one. Also the intuitive interaction of “select, transfer, and go” may enhance the content migration experience relative to distant interaction techniques where users aiming camera for QR-codes [27, 28], or waving the device for device identification [24].

### System stability security issues

The proposed method has potential issues regarding system stability and security. We have demonstrated the system stability in number of experimental conditions of the number of users, and environmental settings. According to the current system setting described in the Support for Simultaneous Multi-User Interaction section, the system is capable of holding 3^*n*−2^ simultaneous users. Therefore, as the number of IPID n increases, the greater number of simultaneous input is possible. However, if the simultaneous inputs exceed this limit, the risk of error increases.

The simultaneous input capability can also increase as the display brightness increases and/or the illuminance sensor’s sensibility and speed increases. The stability in terms of device binding success rate may also be dependent on the environmental context. We have reported a precise quantified results on this parameters as a guideline to guarantee the stability.

In case of the server failure, both the public display and the mobile phone won’t be able to provide the binding service. However, if the network failure occurs only between a certain public display and the server, it will not influence the stability of the remaining displays as well as the mobile phones. The stability maintenance mechanisms should be supported to manage such potential failures.

Regarding the security, the current system could be fragile to attacks that snatches the server connection on either on of the public display side or the mobile device (e.g., man in the middle attack). In case of the attack on the public display side, it may deliver a compromised IPID, that would be interpreted as an unintended content URI or may even not be able to be interpreted. This particular issue would cause inconvenience that either doesn’t return the content the user intended or cause a binding failure (if the IPID does not exist). If the mobile side application is hacked and is connected to a compromised server, the issue may be more severe. Based on the illuminating pattern, the mobile side application may direct the user to a compromised web content, possibly another malware. These security issues can partly be resolved with the secure connections using HTTPS, to prevent from malicious packet snatching.

## Conclusion

In this paper, we proposed illuminance sensor based public display interaction method including the illumination pattern generation and sensing algorithm, its performance evaluation in different environmental settings, and a comparative evaluation with other existing interaction types. Our method is not perfect in every aspect, but does not require additional hardware support to implement. It is robust under general interaction environments except for the extreme outdoor interactions. The overall performance in interaction completion time and error rate was similar or superior than the existing methods.

However, there are several limitations. First, our method inevitably has a binding delay. In order to accurately acquire the illuminance sensor data, the IPID signaling requires the displayed content to change its brightness with the minimum interval of 180*ms*. This delay is due to the illuminance sensor capability which we expect to be improved in the future. Also we had relatively a small number of participants for comparative evaluation. Thereby the subjectivity related to individual performances could have influenced the results. This will also be looked into by expanding the participants in the future study.

We expect both researchers and practitioners in the inter-device interaction domain as well as the digital public signage service providers to benefit from our work.
